# Maternal morbidity and perinatal outcome in preterm premature rupture of membranes before 37 weeks gestation

**DOI:** 10.12669/pjms.303.4853

**Published:** 2014

**Authors:** Saira Dars, Safia Malik, Irum Samreen, Roshan Ara Kazi

**Affiliations:** 1 Saira Dars, MS Obstetrics & Gynaecology, Department of Obstetrics & Gynaecology, Liaquat University Hospital, Hyderabad, Sindh, Pakistan.; 2 Safia Malik, Postgraduate Scholar Obstetrics & Gynaecology, Department of Obstetrics & Gynaecology, Liaquat University Hospital, Hyderabad, Sindh, Pakistan.; 3 Dr. Irum Samreen, Postgraduate Scholar Obstetrics & Gynaecology, Department of Obstetrics & Gynaecology, Liaquat University Hospital, Hyderabad, Sindh, Pakistan.; 4 Prof. Roshan Ara Qazi, Professor Obstetrics & Gynaecology, Liaquat University of Medical & Health Sciences, Jamshoro, Sindh, Pakistan.

**Keywords:** Maternal Morbidity, Perinatal Morbidity, PPROM

## Abstract

**OBJECTIVE: **To assess the maternal morbidity and perinatal outcome in pre-term pre mature rupture of membranes between 24 to 37 weeks gestation.

**METHODS: **This observational study was carried out in Gynaecology & Obstetrics Unit – I, at University Hospital Hyderabad, from October 2010 to October 2011. It included one hundred patients admitted through the outpatient department, as well as from casualty department of University Hospital Hyderabad. Detailed Clinical examination of the patient was done. Systemic review was also done to see any co-morbidity. All patients had laboratory investigations. Inclusion criteria were all patients gestational age between 24 to 37 weeks with preterm premature rupture of membrane (PPROM) confirmed by ultrasound and clinical examination regardless of their age. Exclusion criteria were patients with congenital anomalies, multiple pregnancy, pre-eclampsia & eclampsia, diabetes mellitus, polyhydramnios1 intrauterine growth restriction and placenta abruption. Data was collected using a proforma. Detailed workup including history, general physical examination, abdomen and pelvic examination and relevant specific investigations were noted.

**RESULTS:** Out of 100 patients included in this study Primigravida were 17% and multigravida 83%. There was wide variation of age ranging from a minimum of 20to >40 years. The mean age was 30+ 3.1 years. Mostly patients belonged to the poor class in 72% cases followed by middle class in 21% and upper class 7%. Analysis shows that out of 100 mothers 26% had PROM of <24 hrs duration and 74% had >24 hrs of duration. Maternal outcome in 16 cases of Preterm Premature Rupture of Membrane findings revealed septicemia in 12% cases and Chorioamnionitis in 12% cases. Fetal outcome in 27 cases of preterm premature rupture of membrane revealed prematurity in 5% cases, fetal distress in 4% cases, cord compression in 5% cases, necrotizing enterocolitis in 2% cases, hypoxia in 9% cases and pulmonary hypoplasia in 2% cases.

**CONCLUSION: **Low socioeconomic status is associated with increased neonatal morbidity due to fetal distress, cord compression, necrotizing enterocolitis, hypoxia and pulmonary hypoplasia at the time of delivery. An appropriate and accurate diagnosis of PROM is critical to optimize pregnancy outcome. It is suggested that the timely diagnosis and management of preterm PROM will allow obstetric care providers to optimize perinatal outcome and minimize neonatal morbidity.

## INTRODUCTION

Preterm premature rupture of membrane (PPROM) is defined as rupture of fetal membrane before onset of labor at less than 37 completed weeks of gestation.^1^ Incidence of PPROM ranges from 3.0-10.0% of all deliveries. ^2^^, ^^3^^, ^^4^ Preterm PROM complicates approximately 3 percent of pregnancies and leads to one third of preterm births.^5^ Preterm delivery affects one in 10 births, 11% in USA and even greater births in developing countries and causes 40-75% neonatal deaths^(1)^. PPROM is one of the important causes of preterm birth that can result in high perinatal morbidity and mortality along with maternal morbidity.^6^

There are numerous risk factors for PPROM, such as intrauterine infection at early gestational age, lower socioeconomic status of pregnant women, inadequate prenatal care and inadequate nutrition during pregnancy, sexually transmitted infections, vaginal bleeding, and smoking during pregnancy.^7^^,^^8^^,^^9^ Both mother and fetus are at greater risk of infection after PPROM.

The fetal and neonatal morbidity and mortality risks are significantly affected by severity of oligohydramnious, duration of latency, and gestation at PROM. The primary complication for the mother is risk of infection. Complications of PROM for the fetus and newborn consist of prematurity, fetal distress, cord compression, deformation and altered pulmonary development leading to pulmonary hypoplasia and pulmonary hypertentsion, necrotizing enterocolitis (NEC) and neurologic disorder. Infectious morbidities in mother, fetus and newborn have been related to both PROM and prolonged rupture of membranes.^3^^, ^^10^

The aim of this study was to evaluate the maternal and prenatal complications in pre term pre mature rupture of membranes between 24 to 37 weeks gestation.

## METHODS

This observational study was carried out in department of Obstetrics and Gynaecology Unit-I at Liaquat University Hospital Hyderabad, from October 2010 to October 2011. Patients admitted in Obstetrics and Gynaecology Unit-III through outpatient & casualty department with gestational age between 24 to 37 weeks with preterm premature rupture of membrane (PPROM) confirmed by ultra sound and clinical examination were included regardless of their age. To collect data proforma was filled in all cases. After admission, detailed workeup including history, general physical examination, abdomen and pelvic examination and relevant /specific investigations were noted. Data were analyzed through SPSS software version 16.This study was initiated after taking permission from ethical review committee (ERC).

## RESULTS

This study was conducted on one hundred patients. Of this most patients 72 belonged to poor class, 21 belonged to middle class and upper class accounted for seven patients.

**Fig.1:**
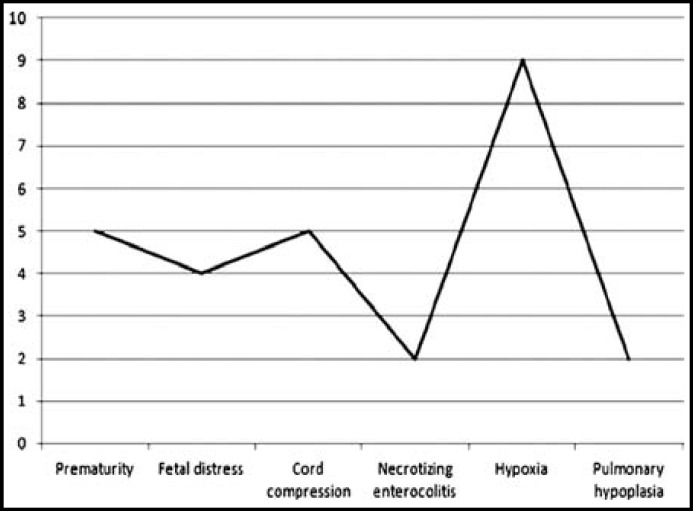
Fetal outcome.

Fetal outcome in 27 cases of preterm premature rupture of membrane revealed prematurity in 5 cases, fetal distress in 4 cases, cord compression in 5 cases, necrotizing enterocolitis in 2 cases, hypoxia in 9 cases and pulmonary hypoplasia in two cases. Fig-1.

## DISCUSSION

Premature rupture of membranes is a fairly common complication of pregnancy and can lead to increased maternal complications, operative procedures, neonatal morbidity and mortality. Prelabor rupture of the membranes occurs in 2% of all births and 40% of all preterm births ^11^^, ^^12^^ , ^^13^. When prelabor rupture of the membranes occurs at term (PROM) there is good evidence that early delivery is associated with a lower incidence of maternal infection and increased maternal satisfaction compared with expectant management ^14^. PPROM near term is a management dilemma. Following membrane rupture the preterm fetus is at risk of a number of complications such as prematurity, placental abruption, ascending infection, intrapartum fetal distress and cord prolapsed ^14^^,^^15^^, ^.

In our study 17 were Primigravida and 83 patients were multigravida . There was wide variation of age ranging from a minimum of 30 years to 45 years. The mean age was 30+ 3.1 years. However it was reported that ^16^ results showed 85 patients were presented with preterm premature rupture of membranes. Out 85 patients 50 were in 15–25 years group, 20 patients in 26–35 years groups and 15 patients in 36–45 years group.

In the present study 72 patinets belonged to poor class, 21% belonged to middle class and upper class accounted for 7%. However in the previous study ^16^ out of 85 patients 58 were from low socioeconomic group, 25 from middle socioeconomic group and two patients wee from the higher socioeconomic group.

The analysis shows that out of 100 mothers 26 had PROM of <24 hrs duration and 74 had >24 hrs of duration. Incidence of documented sepsis in the neonates born to mothers with rupture of membranes greater than 24 hours is approximately 1%. When signs and symptoms of chorioamnionitis are present the risk of proven sepsis increases to 3% to 5% ^17^. When prolonged rupture of membranes is accompanied with prematurity, the incidence of proven sepsis is 4-6% and in highly suspected and proven sepsis the rate is 7- 11% ^18^. In our study maternal outcome in 16 cases of Preterm premature rupture of membrane was septicemia in 12 cases and Chorioamnionitis in 12(12%) cases.

Patients with preterm PROM the most likely outcome is preterm delivery within one week with its associated morbidity and mortality risk such as respiratory distress necrotising enterocolitis, intra ventricular haemorrhage and sepsis ^19^. The incidence of neonatal infection for infants born to women with PROM range from 1–2.6% ^20^. In many studies it was found that the risk of neonatal infection was increased among mother colonised with group B streptococci, premature rupture of membranes >18 hours maternal fever during labour and prematurity ^21^.

In our study fetal outcome in 27 cases of preterm premature rupture of membrane revealed prematurity in 5 cases, fetal distress in 4 cases, cord compression in 5 cases, necrotizing enterocolitis in 2 cases, hypoxia in 9 cases and pulmonary hypoplasia in 2 cases.

Fetal morbidity after preterm PROM results from maternal intrauterine infection, umbilical cord compression, placental abruption, and prolonged fetal compression is caused by oligohydramnios. In each of these places the fetus is at increased risk of fetal death (generally approximately 1% with conservative management after the limit of potential neonatal viability) and perinatal asphyxia ^22^.

The pregnancy complicated by PROM before the limit of fetal viability (currently by 23 weeks) is at increased risk for fetal death (15%); however, a portion of this increase is attributed to non-intervention for fetal benefit when delivery occurs before there is no hope of postnatal survival. When membrane rupture occurs well before the limit of fetal viability (particularly when there is persistent oligohydramnio), there is a significant risk of lethal fetal pulmonary hypoplasia caused by arrested alveolar development. This becomes evident with failure of lung growth despite prolonged latency. Prolonged compression can lead to fetal restriction deformities, similar to those seen in Potter’s syndrome ^22^^, ^^23^. There is accumulating evidence that in utero exposure to infection increases the risk of long-term neurologic sequlae , although there are no current data to demonstrate that delivery before the onset of clinical symptoms of infection prevents these adverse outcomes ^24^.

## CONCLUSION

The study shows majority of PROM patients were in age group of 20-30 yrs. However no significant correlation was found between age group and occurrence of PROM. It is concluded that there is low socioeconomic status which is associated with increased neonatal morbidity due to fetal distress, cord compression, necrotizing enterocolitis, hypoxia and pulmonary hypoplasia at the time of delivery. An appropriate and accurate diagnosis of PROM is critical to optimize pregnancy outcome. It is suggested that the timely diagnosis and management of preterm PROM will allow obstetric care providers to optimize perinatal outcome and minimize neonatal morbidity
